# Metformin use and survival outcomes in endometrial cancer: a systematic review and meta-analysis

**DOI:** 10.18632/oncotarget.20388

**Published:** 2017-08-22

**Authors:** Weimin Xie, Tianjia Li, Jing Yang, Mengmeng Shang, Ying Xiao, Qian Li, Jiaxin Yang

**Affiliations:** ^1^ Department of Obstetrics and Gynecology, Peking Union Medical College Hospital, Chinese Academy of Medical Sciences and Peking Union Medical College, Beijing, China; ^2^ Department of Vascular Surgery, Peking Union Medical College Hospital, Chinese Academy of Medical Sciences and Peking Union Medical College, Beijing, China; ^3^ Department of Obstetrics and Gynecology, Maternal and Child Health Care Hospital of Hunan Province, Changsha, China; ^4^ Department of Scientific Research, Peking Union Medical College Hospital, Chinese Academy of Medical Sciences and Peking Union Medical College, Beijing, China; ^5^ Department of Pathology, Peking Union Medical College Hospital, Chinese Academy of Medical Sciences and Peking Union Medical College, Beijing, China; ^6^ Department of Endocrinology, Key Laboratory of Endocrinology, Ministry of Health, Peking Union Medical College Hospital, Chinese Academy of Medical Sciences and Peking Union Medical College, Beijing, China

**Keywords:** metformin, endometrial cancer, survival outcomes, meta-analysis

## Abstract

Previous studies have evaluated the effects of metformin use on survival outcomes in endometrial cancer, but their results are inconsistent. We conducted a systematic review and meta-analysis to provide a quantitative assessment of the drug's effects based on available evidence. We searched PubMed, Embase, and the Cochrane Central Register of Controlled Trials to identify relevant studies that evaluated the association between metformin use on survival outcomes in endometrial cancer. Pooled hazard ratios (HRs) with 95% confidence intervals (CIs) were calculated to evaluate the association of metformin use with overall survival and with progression-free survival using a fixed-effects model. A total of nine studies involving 2,016 patients with endometrial cancer were identified. In a meta-analysis of eight studies involving 1,594 individuals, metformin use was associated with significant improvements in overall survival (HR, 0.51; 95% CI, 0.41 to 0.64). Metformin users similarly showed improved progression-free survival in a meta-analysis of two studies involving 632 individuals (HR, 0.63; 95% CI, 0.46 to 0.87). In conclusion, endometrial cancer patients who use metformin show improved overall survival and progression-free survival. Further studies are required to confirm the full potential effects of metformin use on survival outcomes in endometrial cancer.

## INTRODUCTION

Metformin is the most widely prescribed drug worldwide for the treatment of type 2 diabetes. It can reduce plasma glucose levels by enhancing insulin sensitivity, which benefits patients with a variety of insulin-resistant states, including impaired glucose tolerance, polycystic ovary syndrome, obesity, and metabolic syndrome [1, 2]. Recent evidence has suggested that metformin may have anti-cancer effects. An increasing number of experimental studies has shown that metformin can effectively inhibit cell proliferation and increase chemo-sensitivity [3, 4]. Observational studies have also found that metformin use is associated with improved survival outcomes in several cancers, including prostate, pancreatic, and colorectal cancer [5–7].

Endometrial cancer is the most common cancer of the female reproductive tract in the developed countries [8]. Given that patients with advanced-stage or recurrent endometrial cancer present with a poor prognosis, it is imperative to investigate the potential factors that are related with survival outcomes in the disease. Obesity, diabetes and insulin resistance have been shown to facilitate the progression of endometrial cancer, and to be strongly associated with a poor prognosis [9, 10]. Experimental studies have demonstrated that metformin has an anti-tumorigenic potential in endometrial cancer cell lines via inhibiting cell growth and decreasing invasion and metastasis [11, 12]. Recently, several studies have tried to evaluate the effects of metformin use on survival outcomes in endometrial cancer; however, their findings were inconsistent. Some studies have shown that metformin use may be associated with improved survival in endometrial cancer [13–15], whereas others have not shown this beneficial effect [16, 17].

To better understand this issue, we carried out a systematic review and meta-analysis of the existing studies that investigated the association between metformin use and survival outcomes in patients with endometrial cancer.

## RESULTS

### Study selection

**Figure 1:**
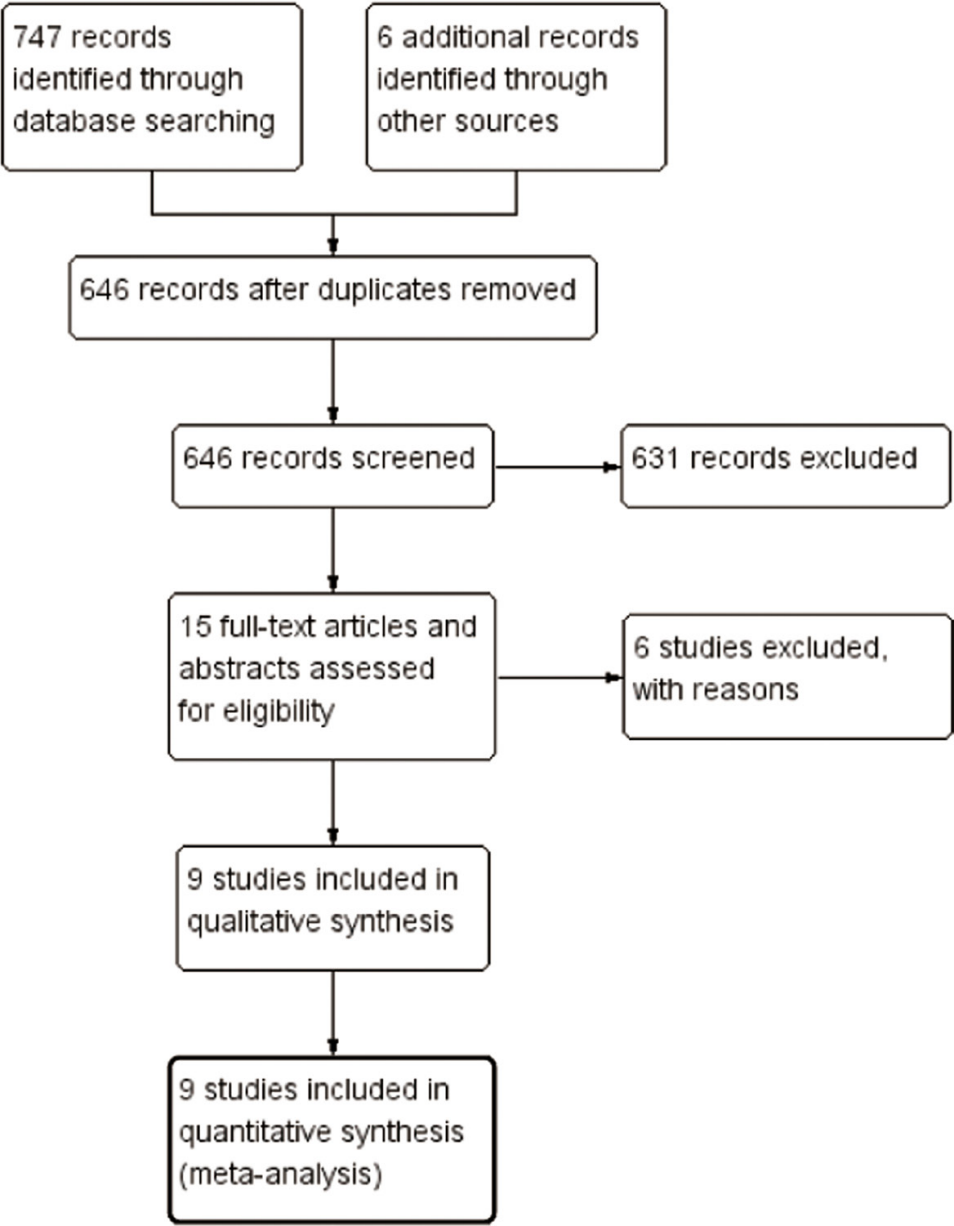
Study flow diagram

After screening 753 reports and conference abstracts, seven full publications and eight conference abstracts were assessed for eligibility. Of these, four records were excluded due to having no available data [18–21], and two were excluded for using overlapped data [22, 23]. Finally, six full publications and three conference abstracts were deemed to meet the eligibility criteria, and were included in the systematic review [13–17, 24–27]. A flow diagram describing study selection is shown in Figure 1.

### Description of studies

A summary of the main characteristics of the included studies is shown in Table 1. All of the included studies were retrospective cohort studies, involving a total of 2,016 patients with endometrial cancer. The nine studies included in the systematic review were published between 2012 and 2016. Of these, seven studies were performed in the United States [13–16, 24–26], one in Poland [17], and one in Austria [27]. Six studies [14–16, 24–26] provided adjusted hazard ratio (HR) estimates, and one [17] presented a crude HR estimate in univariate analysis. Two studies [13, 27] did not provide the HRs and 95% confidence intervals (CIs), the crude HR estimates were calculated from the corresponding Kaplan-Meier curves. The Newcastle–Ottawa scale (NOS) values for the included cohort studies are shown in Table 1. All six studies were considered to be of high-quality: four studies [15, 16, 25, 26] were awarded nine stars, and two studies [17, 27] were awarded seven stars.

**Table 1:** Characteristics of included studies

First author	Study location	Study design	Study population	Stage	Primary treatment (s)	No. of patients	No. of patients on metformin	Outcomes of interest	Adjustments*	NOS value
Lin et al. 2012 [24]	USA	Retrospective cohort study	EAC patients	I–IV	Surgery ± radiotherapy ± chemotherapy	422	22	DFS	1–3	–
Hahn et al. 2014 [13]	USA	Retrospective cohort study	EC patients with DM	I–IV	NA	97	51	OS	–	–
Pierce et al. 2014 [14]	USA	Retrospective cohort study	EC patients with DM	NA	NA	494	282	OS, PFS	1, 2, 4–6	–
Ko et al. 2014 [15]	USA	Retrospective cohort study	EC patients with DM	I–IV	Surgery ± radiotherapy ± chemotherapy	363	200	OS, RFS	1, 2, 4, 6, 7	9
Nevadunsky et al. 2014 [16]	USA	Retrospective cohort study	EC patients with DM	I–IV	Surgery ± radiotherapy ± chemotherapy	250	114	OS	1, 2, 4, 6, 8	9
Lemańska et al. 2015 [17]	Poland	Retrospective cohort study	EC patients	I–III	Surgery ± radiotherapy ± chemotherapy	107	30	OS	–	7
Al Hilli et al. 2016 [25]	USA	Retrospective cohort study	EC patients with DM	I–IV	Surgery ± radiotherapy ± chemotherapy	138	69	OS, PFS	9	9
Ezewuiro et al. 2016 [26]	USA	Retrospective cohort study	EC patients with DM	III, IV or recurrent	Chemotherapy	58	31	OS	1, 4, 10	9
Seebacher et al. 2016 [27]	Austria	Retrospective cohort study	EC patients with DM	I–IV	Surgery ± radiotherapy ± chemotherapy	87	46	OS	–	7

Abbreviations: EAC, endometrioid adenocarcinoma; EC, endometrial cancer; DM, diabetes mellitus; DFS, disease-free survival; OS, overall survival; PFS, progression-free survival; RFS, recurrence-free survival; NOS, Newcastle-Ottawa scale.

* 1, stage; 2, grade; 3, lymphovascular invasion; 4, age; 5, body mass index; 6, treatment; 7, histology; 8, hyperlipidemia; 9, propensity score; 10, study site.

### Meta-analysis

#### Overall survival (OS)

**Figure 2:**
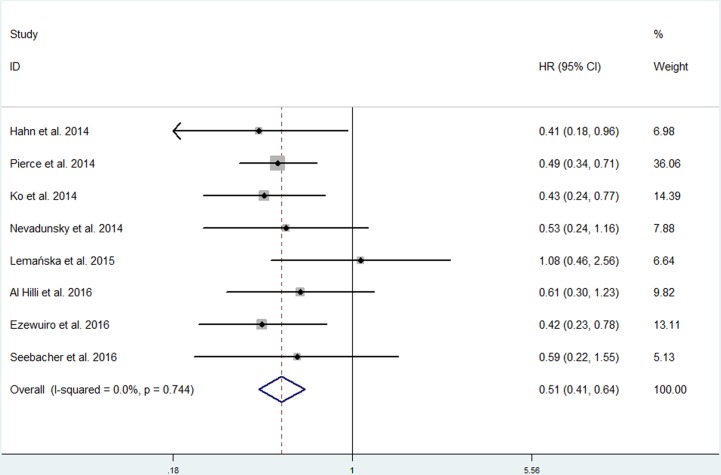
Forest plot of the effect of metformin use on overall survival in endometrial cancer patients

Eight studies involving 1,594 individuals investigated the association between metformin use and OS [13–17, 25–27]. Four studies observed a significant inverse association between metformin use and OS [13–15, 26], while the other four studies did not [16, 17, 25, 27]. There was no substantial heterogeneity among the studies (*p* = 0.744; *I*^2^ = 0.0%). When we pooled the results using a fixed-effects model, metformin use was associated with significant improvements in OS (HR, 0.51; 95% CI, 0.41 to 0.64) (Figure 2).

**Figure 3:**
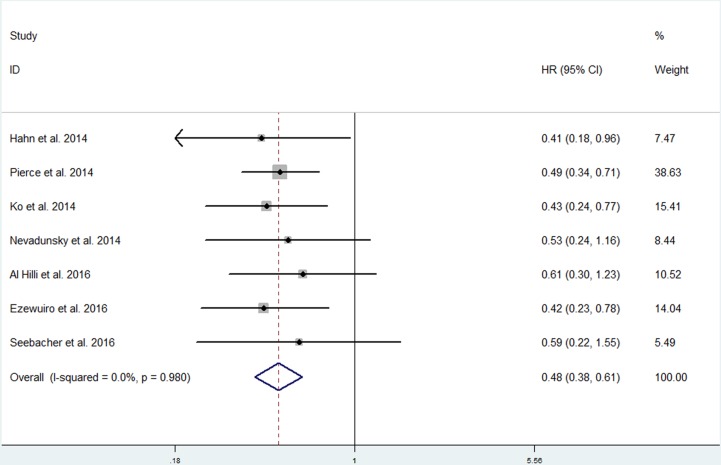
Forest plot of the effect of metformin use on overall survival in in endometrial cancer patients with diabetes

Seven studies involving 1,487 individuals investigated the association between metformin use and OS in endometrial cancer patients with diabetes [13–16, 25–27]. There was no substantial heterogeneity among the studies (*p* = 0.980; *I*^2^ = 0.0%). The pooled data obtained using a fixed-effects model showed an improved OS for metformin users over non-users among endometrial cancer patients with diabetes (HR, 0.48; 95% CI, 0.38 to 0.61) (Figure 3).

To assess the stability of the results, we also conducted sensitivity analysis. In this analysis, the HR and 95% CI did not change significantly after removing any one study, confirming the stability of our results.

### Progression-free survival (PFS)

**Figure 4:**
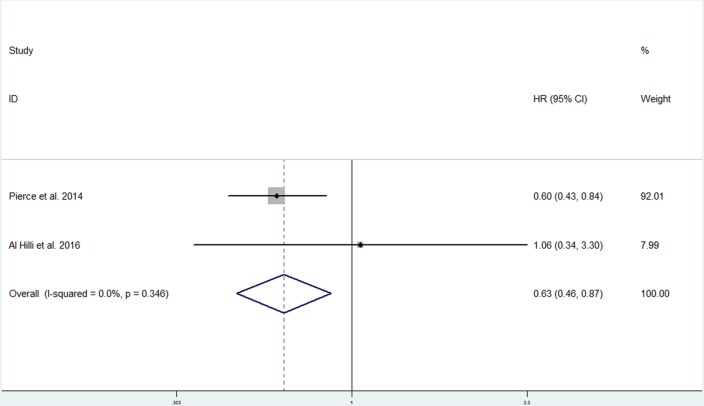
Forest plot of the effect of metformin use on progression-free survival in endometrial cancer patients

Two studies involving 632 individuals explored the association between metformin use and PFS [14, 25]. There was no substantial heterogeneity among the studies (*p* = 0.346; *I*^2^ = 0.0%). The pooled data obtained using a fixed-effects model showed that metformin use was significantly associated with improved PFS (HR, 0.63; 95% CI, 0.46 to 0.87) (Figure 4).

### Data not included in the meta-analysis

As data on either recurrence-free survival (RFS) or disease-free survival (DFS), respectively, was available in a single study, no meta-analysis was performed for these outcomes. One retrospective cohort study containing 363 diabetic endometrial cancer patients evaluated the effect of metformin use on RFS [15]. The result showed that non-metformin users was associated with decreased RFS compared to metformin users, in the unadjusted (HR: 1.7; 95% CI, 1.3 to 2.6) or adjusted model (HR: 1.8; 95% CI, 1.1 to 2.9), when adjusting for age, stage, grade, histology, and adjuvant treatment. The other retrospective cohort study containing 422 patients with endometrioid adenocarcinoma evaluated the effect of metformin use on DFS [24]. The result showed that metformin use was associated with significantly improved DFS (HR: 0.65; 95% CI, 0.43 to 0.99), after adjusting for stage, grade, and lymphovascular invasion.

## DISCUSSION

With a poor prognosis associated with advanced-stage or recurrent disease, the identification of potential adjuvant agents for endometrial cancer is highly desirable. After a systematic search of published and unpublished evidence, the present systematic review identified nine cohort studies with a total of 2,016 individuals that addressed the association between metformin use and survival outcomes in endometrial cancer. Our analysis shows that metformin use is associated with improved OS and PFS in the treatment of endometrial cancer patients. Similarly, our results support the hypothesis that metformin users have longer survival as compared with metformin non-users among endometrial cancer patients with diabetes.

Experimental studies have suggested that metformin can exert anti-neoplastic effects in endometrial cancer cells. *In vitro* cell system analyses have demonstrated that metformin can inhibit cell proliferation, induce apoptosis, attenuate invasion and metastasis, reverse progestin resistance, and enhance chemosensitivity to cisplatin and paclitaxel [11, 12, 28–30]. The mechanisms that underlie the antineoplastic effects of metformin are incompletely understood. Directly, metformin reduces mitochondrial oxidative phosphorylation and ATP production, resulting in an increased AMP:ATP ratio, which leads to activation of the liver kinase B1 (LKB1)-dependent AMP-activated protein kinase (AMPK) pathway [31–33]. AMPK activation leads to the regulation of multiple signaling pathways that control cell proliferation, including down-regulation of the mTOR and the IGF-1/AKT pathways, and in p53-mediated cell-cycle arrest [34–36]. Indirectly, metformin reduces insulin resistance, resulting in a reduction of circulating glucose and insulin levels, which may able to inhibit tumor growth [33, 35].

Consistent with the results from laboratory studies, the present systematic review adds to the growing body of evidence supporting the concept that metformin may have therapeutic potential in endometrial cancer. Our findings are in line with the meta-analyses on the relationship between metformin use and other cancers. Similarly, they found that metformin use was associated with favorable survival outcomes for patients with kidney, pancreatic, liver, and lung cancer [37–40]. A recently published meta-analysis of six studies also found that metformin users had an improved survival over non-users among endometrial cancer patients (HR, 0.63; 95% CI, 0.45–0.87) [41].

Compared with the recently published study [41], our systematic review has several advantages. In addition to electronic databases, we also searched conference abstracts and clinical trial registers for all relevant evidence. No language restrictions were applied in our search strategy. As a result, we identified a larger number of studies as well as a larger sample size than the prior study did, which may result in more reliable findings. We considered more outcomes of interest, including OS, PFS, DFS, and RFS. Besides the consistent findings on OS, we also pooled the data on PFS and found that metformin use was significantly associated with improved PFS. Given that the observed improved OS may partly be due to the cardiovascular protective effects of metformin, the improvement in PFS identified further confirms a direct anti-neoplastic effect of metformin.

Nevertheless, our systematic review had some limitations. First, the available data was only derived from retrospective cohort studies, which may be more susceptible to bias than RCTs, due to their study design. Second, the adjusted confounders were not the same among the six studies, while no adjusted HR estimates were provided in the remaining three studies. Third, the number of included studies was small, and most of them did not report on cancer recurrence or progression outcomes, including PFS, DFS, and RFS. In addition, the dose-response analyses were not performed because most studies did not provide data on the relationship between the frequency, dose, and duration of metformin use and survival outcomes in endometrial cancer.

In conclusion, the present systematic review and meta-analysis demonstrates that metformin use is associated with improved OS and PFS in endometrial cancer patients. These findings add to the laboratory and observational data indicating that metformin may be a useful adjuvant agent in endometrial cancer. Considering the inherent biases of observational studies and the limited data, the results of this systematic review should be interpreted with caution. The full potential effect of metformin use on survival outcomes in endometrial cancer should be further rigorously accessed through randomized trials in the future.

## MATERIALS AND METHODS

This systematic review and meta-analysis was prepared according to the Preferred Reporting Items for Systemic Reviews and Meta-Analyses (PRISMA) Statement [42].

### Search strategy

We searched the databases of PubMed, Embase, and the Cochrane Central Register of Controlled Trials (CENTRAL) to identify relevant studies from their inception to May 24, 2017. The detailed search strategies can be found in Supplementary Table 1. We searched the ISRCTN registry, ClinicalTrials.gov, and the World Health Organization International Clinical Trials Registry Platform (ICTRP) for relevant ongoing trials. We also searched reports from the following conferences: the Biannual Meeting of the International Gynecologic Cancer Society, the Biannual Meeting of the European Society of Gynecologic Oncology, the Annual Meeting of the American Society of Clinical Oncology, and the Annual Meeting on Women's Cancer of the Society of Gynecologic Oncology. In addition, we scanned the references of the retrieved articles for additional eligible studies. There were no language restrictions employed in our search strategy.

### Eligibility criteria

The identified potentially relevant articles were evaluated in detail to determine their eligibility. The aim was to evaluate OS, PFS, DFS, and RFS. Retrieved articles had to meet the following inclusion criteria: (1) RCTs or non-randomized studies (cohort or case-control studies) estimating the association between metformin use and survival outcomes of endometrial cancer patients; (2) full-text articles and abstracts that reported HRs and 95% CIs, or provided sufficient data to calculate these. Review articles or letters without original data, editorials, and case reports were excluded. If there were multiple publications involving the same population, the most comprehensive study was included.

### Data extraction and quality assessment

Two reviewers performed data extraction independently. For each article, we collected information about the authors, publication year, study location, study population, tumor stage, sample size, outcomes of interest, HRs and 95% CIs, and variables adjusted in the analysis. The HR estimates that reflected the greatest degree of control for potential confounders were extracted whenever possible. If the HRs and 95% CIs were not available, we calculated them indirectly from Kaplan-Meier curves using published methods [43, 44]. To maintain consistency among the included studies, we extracted the HR estimates that compared metformin users with metformin non-users. When the study population was divided into three groups as non-diabetics, diabetics taking metformin, and diabetics not taking metformin, we only extracted the HR estimate that compared diabetics taking metformin and diabetics not taking metformin, in order to evaluate the effects of metformin on endometrial cancer patients with diabetes.

Since all of the included studies were cohort studies, the methodological quality was evaluated independently by two authors using the NOS [45]. The NOS uses a star system that awards from 0 to 9 stars with respect to three parameters: selection, comparability, and outcome. Since no standard criteria has been established, we considered studies that were awarded seven or more stars to be high quality studies. Any disagreements between the two authors were resolved by discussion or in consultation with a third author.

### Statistical analysis

Pooled HRs with 95% CIs were used to evaluate the association of metformin use with OS and PFS of endometrial cancer patients. Heterogeneity among studies was measured using the Chi-square (χ^2^, or Chi2) test, and quantified using the I^2^ statistic. When substantial heterogeneity (*p* value < 0.10 or *I*^2^ > 50%) was found, pooled HRs were calculated using a random-effects model; otherwise, a fixed-effects model was applied. We planned to conduct sensitivity analyses by removing each individual study from the meta-analysis. Since the number of included studies was fewer than 10 in this meta-analysis, we did not evaluate publication bias [46, 47]. All analyses were conducted using Stata version 12.0 software (Stata Corporation, College Station, TX, USA).

## SUPPLEMENTARY MATERIALS FIGURES AND TABLES


