# Supplementary material for: Metformin use and survival outcomes in endometrial cancer: a systematic review and meta-analysis
Source: Oncotarget. 2017 Aug 22;8(42):73079–86. doi: 10.18632/oncotarget.20388 (PMC5641193; doi:10.18632/oncotarget.20388)
Supplement: Supplementary file 1 [file oncotarget-08-73079-s001.pdf]

Metformin use and survival outcomes in endometrial cancer: a systematic review and meta-analysis

SUPPLEMENTARY MATERIALS

Search strategy

Pubmed, Embase and Cochrane Central Register of Controlled Trials (CENTRAL) were searched from the inception to May 24, 2017, using the following search strategy.

PubMed search strategy:

#1 Metformin [Mesh]
#2 metformin*
#3 dimethylbiguanidine OR dimethylbiguanidium OR dimethylguanylguanidine OR glucophage OR glucovance
#4 #1 OR #2 OR #3
#5 "Endometrial Neoplasms"[Mesh]
#6 endometr*
#7 neoplas* OR cancer* OR carcinom* OR malignan* OR tumor* OR tumour*
#8 #6 AND #7
#9 #5 OR #8
#10 #4 AND #9

Embase search strategy:

#1 'metformin'/exp
#2 metformin*
#3 dimethylbiguanidine OR dimethylbiguanidium OR dimethylguanylguanidine OR glucophage OR glucovance
#4 #1 OR #2 OR #3
#5 'endometrium tumor'/exp
#6 endometr*
#7 neoplas* OR cancer* OR carcinom* OR malignan* OR tumor* OR tumour*
#8 #6 AND #7
#9 #5 OR #8
#10 #4 AND #9

CENTRAL search strategy:

#1 MeSH descriptor: [Metformin] explode all trees
#2 metformin*
#3 dimethylbiguanidine OR dimethylbiguanidium OR dimethylguanylguanidine OR glucophage OR glucovance
#4 #1 OR #2 OR #3
#5 MeSH descriptor: [Endometrial Neoplasms] explode all trees
#6 endometr* near/5 (neoplas* OR cancer* OR carcinom* OR malignan* OR tumor* OR tumour*)
#7 #5 OR #6
#8 #4 AND #7
